# Predictive Performance of Artificial Intelligence Algorithms for Gestational Diabetes Mellitus in Pregnant Women: Systematic Review and Meta-Analysis

**DOI:** 10.2196/79729

**Published:** 2026-01-30

**Authors:** Yingni Liang, Anran Dai, Meiyan Luo, Zhuolian Zheng, Jiayu Shen, Yinhua Su, Zhongyu Li

**Affiliations:** 1 School of Nursing, University of South China, No. 28, Changsheng West Road, Hengyang, Hunan, 421001, China, 867348281809; 2 Department of Obstetrics, Second Affiliated Hospital of the University of South China, Hunan, China

**Keywords:** gestational diabetes mellitus, artificial intelligence, prediction, meta-analysis, PRISMA, Preferred Reporting Items for Systematic Reviews and Meta-Analysis

## Abstract

**Background:**

Gestational diabetes mellitus (GDM) is a common complication during pregnancy, with its incidence increasing year by year. It poses numerous adverse health effects on both mothers and newborns. Accurate prediction of GDM can significantly improve patient prognosis. In recent years, artificial intelligence (AI) algorithms have been increasingly used in the construction of GDM prediction models. However, there is still no consensus on the most effective algorithm or model.

**Objective:**

This study aimed to evaluate and compare the performance of existing GDM prediction models constructed using AI algorithms and propose strategies for enhancing model generalizability and predictive accuracy, thereby providing evidence-based insights for the development of more accurate and effective GDM prediction models.

**Methods:**

A comprehensive search was conducted across PubMed, Web of Science, Cochrane Library, EMBASE, Scopus, and OVID, covering publications from the inception of databases to June 1, 2025, to include studies that developed or validated GDM prediction models based on AI algorithms. Study selection, data extraction, and risk of bias assessment using the Prediction Model Risk of Bias Assessment Tool were performed independently by 2 reviewers. A bivariate mixed-effects model was used to summarize sensitivity and specificity and to generate a summary receiver operating characteristic (SROC) curve, calculating area under the curve (AUC). The Hartung-Knapp-Sidik-Jonkman method was further used to adjust for the pooled sensitivity and specificity. Between-study standard deviation (τ) and variance (τ²) were extracted from the bivariate model to quantify absolute heterogeneity. The Deek test was used to evaluate small-study effects among included studies. Additionally, subgroup analysis and meta-regression were conducted to compare the performance differences among algorithms and to explore sources of heterogeneity.

**Results:**

Fourteen studies reported on the predictive value for AI algorithms for GDM. After adjustment with the Hartung-Knapp-Sidik-Jonkman method, the pooled sensitivity and specificity were 0.78 (95% CI 0.69‐0.86; *τ*=0.15, τ^2^=0.02; PI 0.47‐1.09) and 0.85 (95% CI 0.78‐0.92; *τ*=0.11, τ^2^=0.01; PI 0.59‐1.11), respectively. The SROC curve showed that the AUC for predicting GDM using AI algorithms was 0.94 (95% CI 0.92‐0.96), indicating a strong predictive capability. Deek test (*P*=.03) and the funnel plot both showed clear asymmetry, suggesting the presence of small-study effects. Subgroup analysis showed that the random forest algorithm exhibited the highest sensitivity (0.83, 95% CI 0.74‐0.93), while the extreme gradient boosting algorithm exhibited the highest specificity (0.82, 95% CI 0.77‐0.87). Meta-regression further revealed an evaluation in predictive accuracy in prospective study designs (regression coefficient=2.289, *P*=.001).

**Conclusions:**

Unlike previous narrative reviews, this systematic review innovatively provided a comparative and quantitative synthesis of AI algorithms for GDM prediction. This established an evidence-based framework to guide model selection and identified a critical evidence gap. The key implication for real-world application was the demonstrated necessity of local validation before clinical adoption. Therefore, future work should focus on large-scale, prospective validation studies to develop clinically applicable tools.

## Introduction

Gestational diabetes mellitus (GDM) is one of the most common metabolic disorders during pregnancy, characterized by glucose metabolism abnormalities that first appear during gestation [1]. The incidence of GDM has risen to 15.8% due to factors like increased childbearing age, dietary changes, and pre-pregnancy obesity [2-4]. GDM not only significantly increased the risk of adverse pregnancy outcomes for pregnant women, such as macrosomia, preterm birth, and preeclampsia, but also had a profound impact on the long-term health of their offspring, including an increased risk of developing obesity, type 2 diabetes, and other metabolic disorders in the future [5-7]. Therefore, early prediction and management of GDM could effectively reduce the incidence of GDM and its associated maternal and neonatal complications, thereby optimizing perinatal care and improving long-term health outcomes.

The emergence of artificial intelligence (AI) algorithms in medicine has opened new frontiers for predictive analytics, offering the potential to model complex, non-linear interactions within multidimensional health data [8]. In fields such as oncology, cardiology, and endocrinology, AI-driven prediction models have demonstrated superior discriminative accuracy compared to conventional statistical approaches, largely by capturing subtle patterns and interactions among risk factors that traditional methods might overlook [9-12]. This capability was particularly salient for GDM, a condition influenced by a dynamic interplay of genetic, metabolic, hormonal, and lifestyle factors [13].

Building on this general capability, the application of AI algorithms for the specific task of GDM prediction has gained considerable momentum, with primary attention to 2 domains: machine learning (ML) and deep learning (DL) [14-16]. Commonly used ML algorithms, such as random forest (RF), support vector machine, and extreme gradient boosting (XGBoost), have been applied to structured clinical and biomarker data, while DL algorithms typically use neural networks to exploit high-dimensional inputs, including eHealth records and even image-based data [17]. Despite promising reported accuracies, a critical and persistent challenge is the marked heterogeneity in model performance across different populations and settings [18-20]. The ML model developed by Gallardo et al [21], based on routine early-pregnancy examination data, showed high predictive accuracy in a particular population but performed poorly in other GDM populations due to differences in data characteristics. This discrepancy revealed a severe methodological inconsistency in these studies, such as the lack of standardized data preprocessing, non-uniform validation strategies, and incomplete reporting of performance metrics. This heterogeneity made it difficult to directly compare and integrate the results of different studies.

Consequently, although a growing body of primary studies investigating AI models for GDM prediction, the evidence in this field remained fragmented and methodologically heterogeneous. Currently, for the prediction of GDM, there was still a lack of systematic reviews and meta-analyses that could directly compare multiple AI algorithms head-to-head, quantitatively assess their cross-population applicability, and systematically examine methodological rigor. The majority of existing original studies have developed single-algorithm models and validated them only within mono-ethnic or single-center cohorts [16,17,21,22]. Consequently, clinicians lack the high-level evidence required to determine which algorithm is superior and whether reported accuracies generalize to other settings, which markedly impedes the credible clinical adoption and broader dissemination of AI-based prediction models.

To address these evidence gaps, this systematic review and meta-analysis aimed to quantitatively synthesize the predictive performance of prediction models constructed using AI algorithms across different scenarios for GDM, compare the effectiveness of different AI algorithms, and identify the key factors influencing performance. By providing a rigorous, evidence-based framework for evaluating and comparing AI prediction models in GDM, this systematic review sought to inform the future development of more robust, generalizable, and clinically actionable tools, thereby supporting efforts toward early identification, risk stratification, and personalized management of GDM.

## Methods

### Registration and Protocol

This systematic review adhered to the Preferred Reporting Items for Systematic Reviews and Meta-Analysis (PRISMA) 2020 extended checklist, with extensions for Diagnostic Test Accuracy (PRISMA-DTA) and literature search reporting (PRISMA-S) [23-25]. The protocol was prospectively registered with PROSPERO (International Prospective Register of Systematic Reviews; ID CRD42025645913). And the registration was completed on February 13, 2025, prior to the commencement of data extraction and analysis (Checklist 1).

### Information Sources and Search Strategy

A comprehensive search was conducted across 6 databases, including PubMed, Web of Science, Cochrane Library, Scopus, EMBASE, and OVID, from the inception of each database to June 1, 2025. To enhance the accuracy of the search results and avoid the omission of relevant studies, the research term developed a rigorous search strategy by combining Medical Subject Headings terms, keywords, and synonyms. No previously published search filters were applied so as to maintain a highly sensitive search strategy. Table 1 summarizes the core search concepts and representative terms. And the detailed search strategy is presented in Multimedia Appendix 1. In addition, we also reviewed the reference lists of relevant literature, particularly systematic reviews related to the topic of this study, and conducted additional searches in the electronic databases to minimize the omission of the key literature as much as possible. All searches were conducted under the supervision of an academic librarian.

**Table 1.** Search strategy using the population, Intervention framework for artificial intelligence–based gestational diabetes mellitus prediction studies.

Concept	Key terms (PubMed example)
Population	“Gestational Diabetes Mellitus” OR “Pregnancy-induced Diabetes” OR “GDM” OR “Diabetes in Pregnancy” OR “Maternal Diabetes”
Intervention	“Artificial Intelligence” OR “Machine Learning Algorithms” OR “Deep Learning Algorithms” OR “Ensemble Learning Algorithms”

### Eligibility Criteria

To screen out the original studies relevant to this systematic review from the retrieved literature, detailed inclusion and exclusion criteria were defined (Textbox 1).

Textbox 1. **Inclusion and exclusion criteria**

**Inclusion criteria**

- Studies that conducted among pregnant women with gestational diabetes mellitus (GDM) or those at risk of developing GDM.
- Studies that completely constructed one or more predictive models for predicting GDM.
- Studies that used AI algorithms for the construction of a predictive model.
- Studies published in English.

**Exclusion criteria**

- Reviews, meta-analysis, protocols, letters, conference abstracts, case reports, and animal studies.
- Studies on the predictive accuracy of single-factor predictors.
- Studies only conducted a risk factor analysis without constructing a predictive model.
- Studies did not include any outcome measures for assessing the predictive accuracy of the predictive model.

### Selection and Data Collection Process

Following the completion of the systematic research, all records were imported into the reference management software Endnote 21. After removing duplicate records, 2 reviewers independently examined the titles and abstracts of each study. Studies not reporting AI-based predictive models were discarded. Subsequently, a thorough full-text assessment was conducted for all studies that initially met the criteria, and the reasons for excluding each study were recorded in detail. In the predesigned Excel spreadsheet, data was extracted from studies that qualified based on the inclusion criteria. The extracted information included: characteristics of the study (authors, country, publication year, study design, and sample size), characteristics of the participants (diagnostic criteria for GDM and number of GDM cases), intervention features (model development process, types of AI algorithms used, methods for handling missing data, predictors, and model validation), and study outcomes (assessment of model accuracy). In cases where the information presented in the literature was ambiguous, the researchers would proactively contact the corresponding author to acquire the relevant information. The aforementioned process was independently conducted by 2 authors. Any discrepancies were discussed and resolved with a third author.

### Study Risk-of-Bias Assessment

The Prediction Model Risk of Bias Assessment Tool (PROBAST) was used to assess the risk of bias (ROB) for each study. PROBAST consisted of four domains: participants, predictors, outcomes, and analysis [26]. Based on the responses to the items provided in the PROBAST checklist, a ROB rating (high, low, or unclear) was assigned to each domain. The criteria for assessment were detailed below: (1) the overall ROB was deemed “low” when all domains were classified as “low risk”; (2) the overall ROB was considered “high” if any domain was identified as “high risk”; (3) the overall ROB was determined to be “unclear” when there was at least one domain with an “unclear” rating, while the other domains were classified as “low risk” [26]. The quality assessment was conducted by the same 2 authors who performed the study selection and data extraction. Any disagreements between the 2 authors were resolved through consultation with a third author.

### Effect Measures and Synthesis Methods

Statistical analyses were performed using Stata (version 17.0; StataCorp LLC), R (version 4.2.0; R Development Core Team), and Meta DiSc (version 1.4; Clinical Biostatistics Unit) software. A bivariate mixed-effects model was used to pool sensitivity and specificity, generate a summary receiver operating characteristic (SROC) curve, and calculate area under the curve (AUC). The Hartung-Knapp-Sidik-Jonkman method was further used to adjust the pooled estimates. All results were reported with 95% CI values. Between-study standard deviation (τ) and variance (τ²) were extracted from the bivariate model to quantify absolute heterogeneity. And prediction intervals (PIs) were subsequently computed to estimate the range within which the true sensitivity or specificity of a future study was expected to lie, providing a clinically interpretable measure of real-world dispersion. Moreover, the Fagan nomogram was used to explore the relationship between pretest probability, likelihood ratios (LR), and post-test probability. The LR dot plot, divided into 4 quadrants based on the strength of evidence threshold, was used to determine the exclusion and confirmation of the AI model. Additionally, a bivariate boxplot was drawn to detect heterogeneity caused by threshold effects. And subgroup analysis was used to compare the predictive capabilities of different AI algorithms in GDM prediction. In line with current recommendations for interpreting heterogeneity, we quantified real-world dispersion primarily using the τ, *τ*^2^, and calculated PIs as the key measure of practical uncertainty [27]. The *I*^2^ statistic was considered but not emphasized, given its limited use informing the generalizability of findings compared to PIs [27]. Based on the clinical and methodological characteristics anticipated to cause heterogeneity across studies, a meta-regression analysis was used to explore and explain such heterogeneity. It aimed to uncover potential influencing factors and analyze which variables might account for variations in the effect sizes. And the Deek test was used to evaluate small-study effects among the included studies, with *P*<.05 indicating funnel-plot asymmetry.

### Ethical Considerations

This systematic review and meta-analysis was conducted exclusively with published aggregate data. No individual-level or identifiable participant information was involved. Therefore, informed consent, institutional review board approval, privacy protection, and participant compensation were not applicable.

## Results

### Study Selection and Characteristics of Included Studies

**Figure 1.**
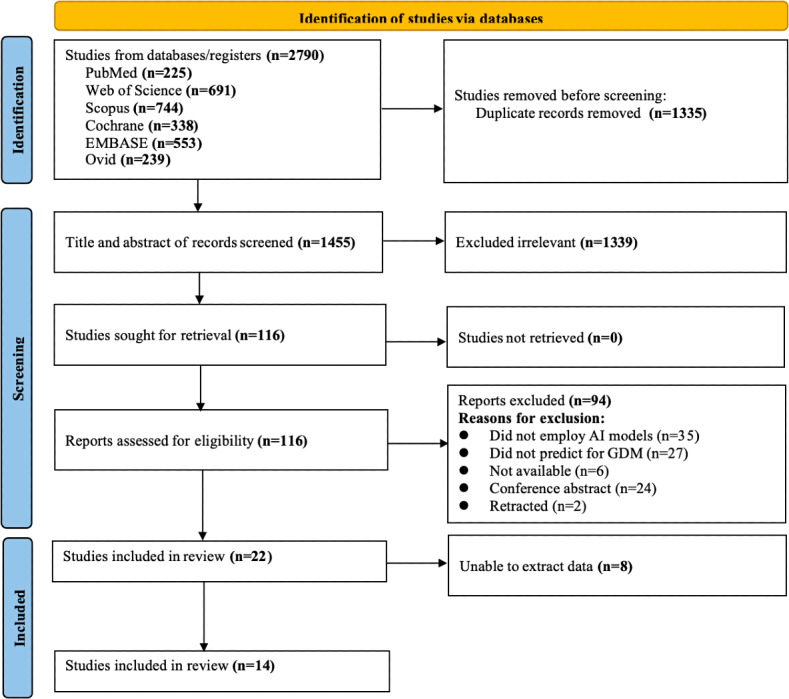
PRISMA (Preferred Reporting Items for Systematic Reviews and Meta-Analysis) flow diagram for study selection. This figure illustrates the process of identifying, screening, and selecting studies for inclusion in the systematic review, showing the number of records at each stage and reasons for exclusions. AI: artificial intelligence; GDM: gestational diabetes mellitus.

A total of 2790 studies were retrieved from the database. After removing duplicates, the titles and abstracts of 1455 studies were reviewed, and the full texts of 116 studies were screened. Finally, 22 studies were included in this study, with 8 studies [14,15,28-35] being included in the systematic review and 14 studies being incorporated into the meta-analysis [15,16,21,22,28,36-44]. The detailed process of the literature screening is illustrated in Figure 1. The fundamental characteristics of the included studies are presented in Table 2. The included studies were conducted in 11 countries, with 12 being single-center studies [14,16,21,28,30-32,36,37,40,41,43], 10 being multicenter studies [15,22,29,33-35,38,39,42,44], 14 being retrospective studies [14,16,21,22,28-30,32,36,37,39-41,43], and 8 being prospective studies [15,31,33-35,38,42,44]. All 22 studies used ML algorithms, and 2 of them further used DL algorithms [16,42]. To evaluate the predictive performance of the models, 12 studies conducted internal validation [14,15,21,22,31,32,34,37,38,40-42], and 8 studies performed external validation [16,28-30,32,37,39,42]. Multimedia Appendix 2 provides a detailed record of the model performance parameters for each study.

**Table 2.** General characteristics of the 22 studies included in the systematic review of artificial intelligence models for gestational diabetes mellitus prediction.

Study	Country	Study type	Single-center or multicenter	Diagnostic criteria	Sample size	Type of model
Belsti et al (2023) [22]	Australia	Retrospective	Multicenter	IADPSG^a^	48,502	ML^b^
Ali et al (2022) [33]	United Arab Emirates	Prospective	Multicenter	IADPSG	3858	ML
Wu et al (2021) [16]	China	Retrospective	Single-center	IADPSG	32,190	ML and DL^c^
Lin and Fang (2023) [36]	China	Retrospective	Single-center	IADPSG	406	ML
Ye et al (2020) [37]	China	Retrospective	Single-center	IADPSG	22,242	ML
Wang et al (2022) [30]	China	Retrospective	Single-center	IADPSG	1075	ML
Wu et al (2021) [28]	China	Retrospective	Single-center	IADPSG	17,005	ML
Wang et al (2021) [38]	China	Prospective	Multicenter	IADPSG	1139	ML
Syngelaki et al (2025) [31]	England	Prospective	Single-center	NICE^d^	41,587	ML
Donovan et al (2019) [39]	America	Retrospective	Multicenter	NIH^e^	11,56,708	ML
Kaya et al (2024) [40]	Turkey	Retrospective	Single-center	IADPSG	97	ML
Hu et al (2023) [41]	China	Retrospective	Single-center	IADPSG	735	ML
Liu et al (2022) [34]	China	Prospective	Multicenter	IADPSG	6848	ML
Lee et al (2021) [42]	Korea	Prospective	Multicenter	NIH	1443	ML and DL
Kumar et al (2022) [35]	Singapore	Prospective	Multicenter	IADPSG	222	ML
Bigdeli et al (2025) [14]	Iran	Retrospective	Single-center	NIH	743	ML
Kurt et al (2023) [15]	Turkey	Prospective	Multicenter	IADPSG	489	DL
Cubillos et al (2023) [21]	Chile	Retrospective	Single-center	IADPSG	1611	ML
Ding et al (2024) [43]	China	Retrospective	Single-center	IADPSG	554	ML
Kang et al (2023) [29]	Korea	Retrospective	Multicenter	NIH	34,387	ML
Zhao et al (2025) [32]	China	Retrospective	Single-center	IADPSG	1,03,172	ML
Liu et al (2020) [44]	China	Prospective	Multicenter	IADPSG	19,331	ML

a IADPSG: International Association of Diabetes and Pregnancy Study Groups.

b ML: machine learning.

c DL: deep learning.

d NICE: National Institute for Health and Care Excellence.

e NIH: National Institutes of Health.

### ROB in Studies

**Figure 2.**
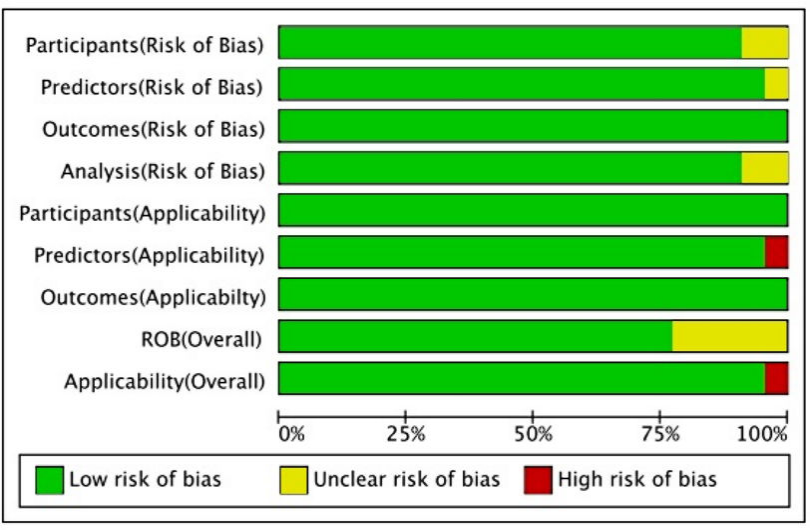
Risk assessment of the included models. This graph summarizes the methodological quality of the included prediction models, categorizing ROB across key domains to help readers assess the reliability of the evidence. ROB: risk of bias

Based on the PROBAST checklist, each study was assessed in terms of participants, predictors, outcomes, and analysis (Figure 2). The majority of studies consistently demonstrated low overall ROB and high applicability, indicating reliable methodology. However, in terms of overall ROB, 5 studies were rated as “unclear” [15,33,35,37,40]. One study was identified as having “high risk” in overall applicability due to insufficiently detailed descriptions of predictors used in model development [15]. Additionally, within the analysis domain, 2 studies were rated as “unclear” due to relatively small sample size, and this might also be one of the potential sources of bias [35,40]. In summary, most studies exhibited strong methodological quality and applicability. The detailed quality assessment of the included studies is detailed in Multimedia Appendix 3.

### Performance of AI Algorithms for GDM

**Figure 3.**
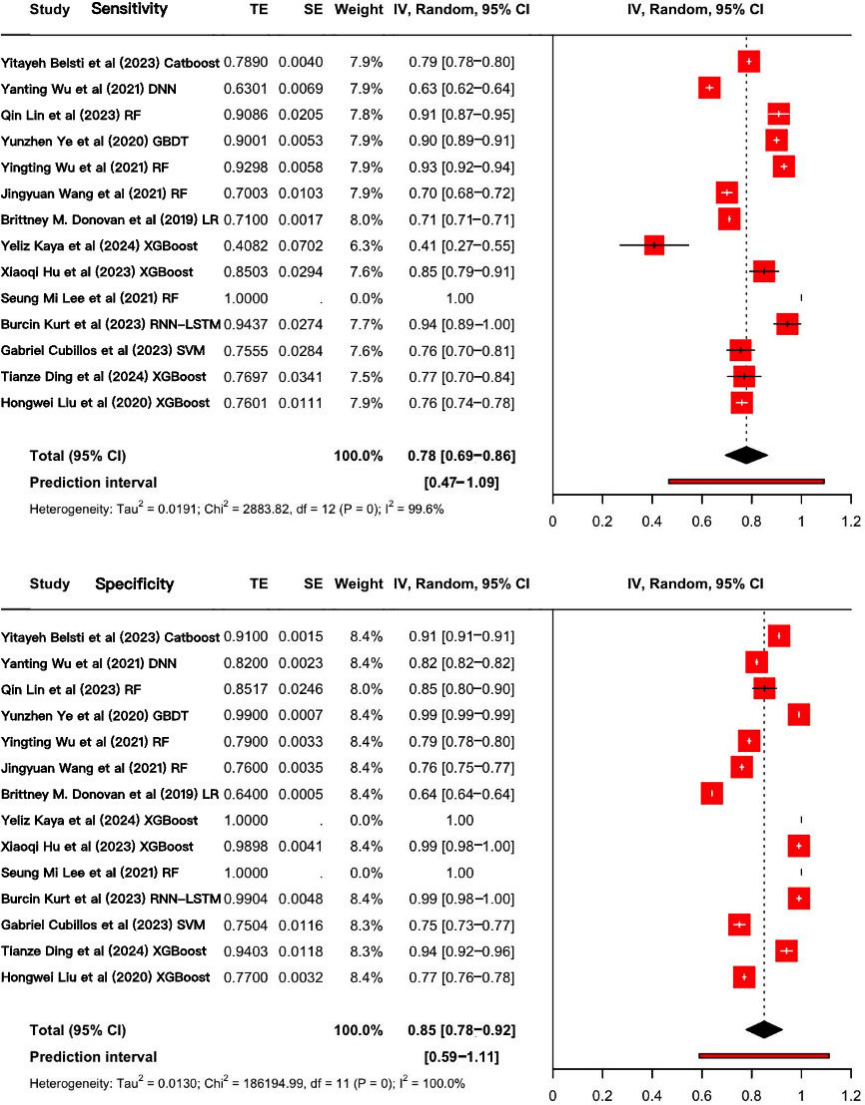
Forest plots of sensitivity and specificity in 14 included studies on using artificial intelligence algorithms for predicting gestational diabetes mellitus. Each horizontal line represents the performance estimate of an individual study, with the diamond indicating the pooled result. The wide variability across studies highlights substantial heterogeneity in model performance [15,16,21,22,28,36-44]. DNN: deep neural network; GBDT: gradient-boosting decision tree; LR: logistic regression; RF: random forest; RNN-LSTM: recurrent neural network-long short-term memory; SVM: support vector machine; XGBoost: extreme-gradient boosting.

A total of 14 studies conducted on independent patient populations were included with the aim of evaluating the predictive value of AI algorithms for GDM [15,16,21,22,28,29,36-43]. Since some studies used multiple AI algorithms to construct several prediction models, this systematic review selected the model with the best performance reported in each study for meta-analysis. The pooled sensitivity was 0.78 (95% CI 0.69‐0.86; *τ*=0.15, τ^2^=0.02; PI 0.47‐1.09), and specificity was 0.85 (95% CI 0.78‐0.92; *τ*=0.11, τ^2^=0.01; PI 0.59‐1.11) after adjustment for the Hartung-Knapp-Sidik-Jonkman method (Figure 3). The wide PIs indicated substantial heterogeneity in real-world performance across populations, supporting the recommendation for local validation in the target population before clinical deployment. Note that the upper bounds of the PIs exceeded 1.0, specifically reaching 1.09 and 1.11. This occurred as a result of back-transformation from the logit scale and was a recognized statistical artifact, which did not indicate actual predictive performance greater than 100%.

**Figure 4.**
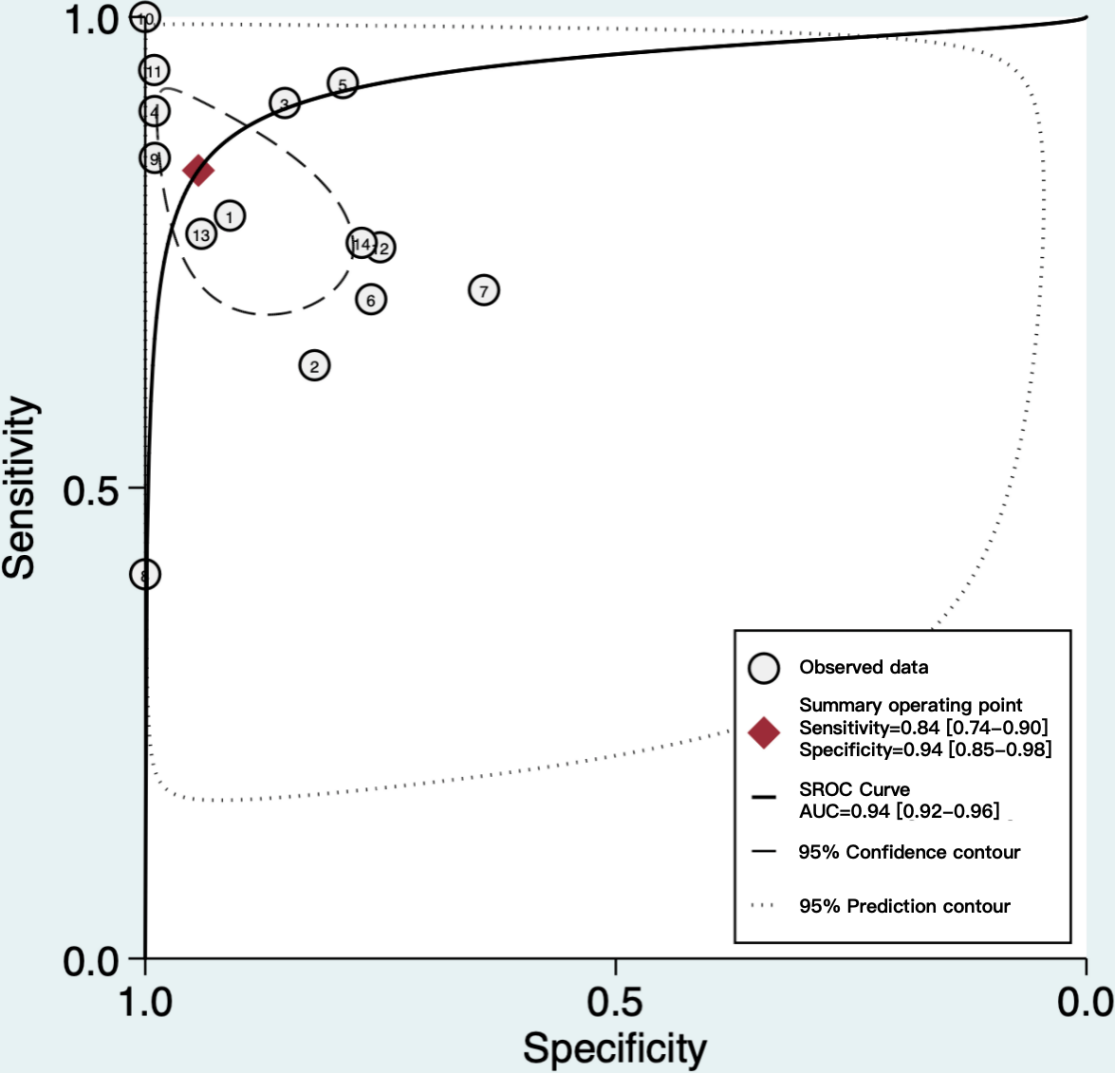
SROCs of included studies. This plot shows the overall diagnostic accuracy of artificial intelligence algorithms, with the curve position indicating the trade-off between sensitivity and specificity across different thresholds. The high AUC (0.87) reflects strong average discriminatory power. SROC: summary receiver operating characteristic curve; AUC: area under the curve.

**Figure 5.**
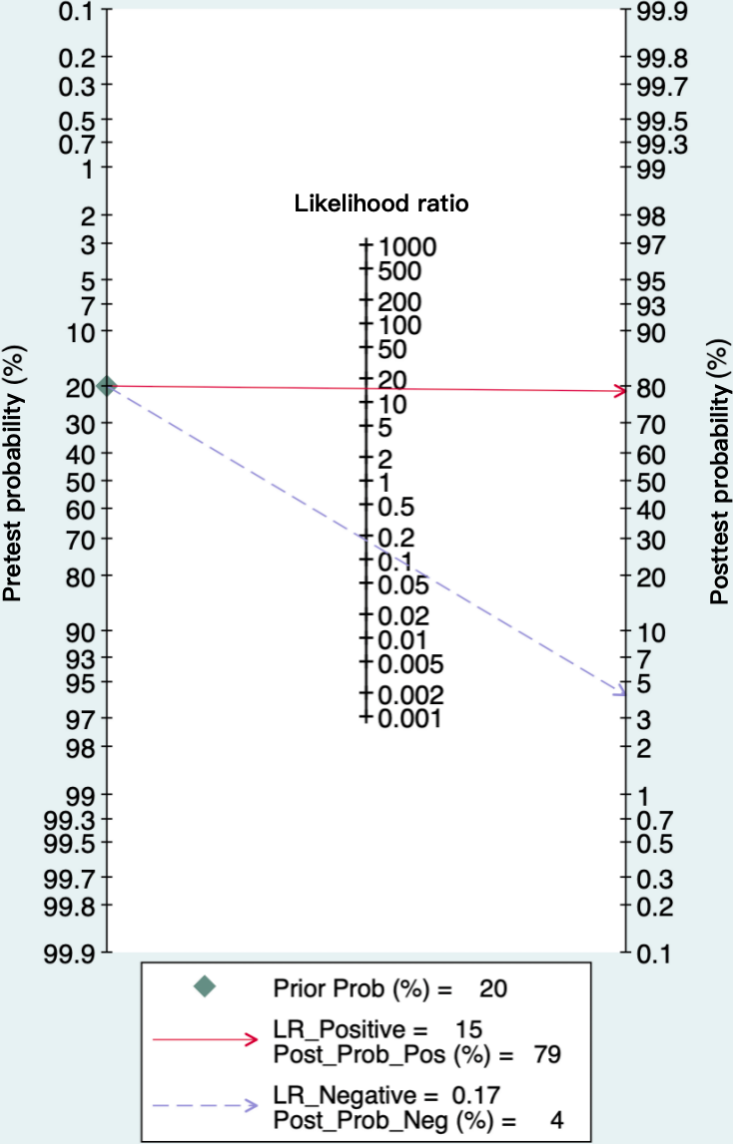
Fagan nomogram of artificial intelligence (AI) algorithms for predicting gestational diabetes mellitus. The first column of this nomogram represents the pretest probability, the second column represents the likelihood ratio, and the third shows the posttest probability. Interpretation: This tool helps clinicians estimate how a positive or negative test result changes the probability of gestational diabetes mellitus. The limited shift from pre to posttest probability indicates that current AI models provide only modest diagnostic value in clinical practice.

**Figure 6.**
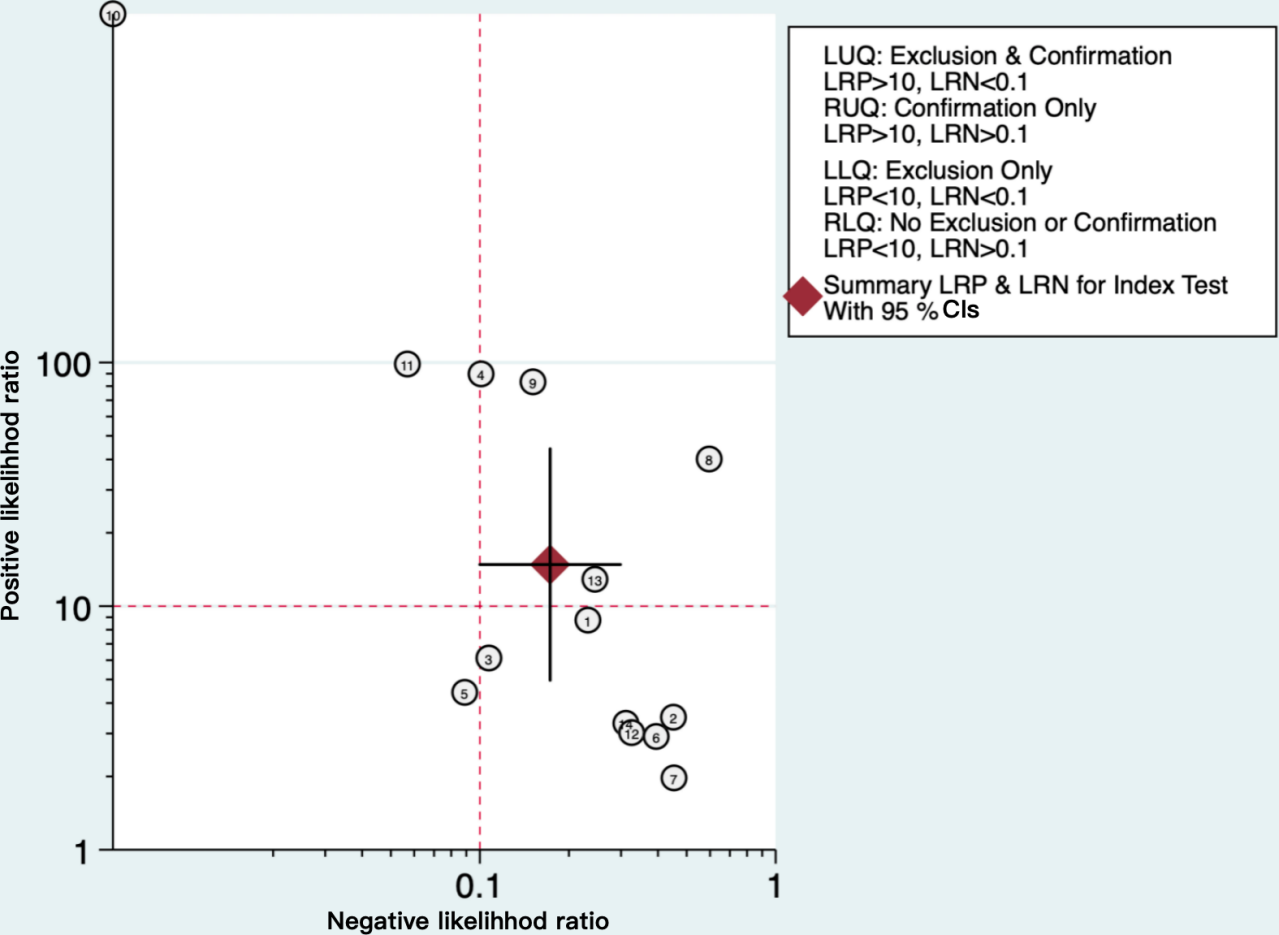
Likelihood ratio dot plot of artificial intelligence algorithms for predicting gestational diabetes mellitus. The position of the summary point in the upper right quadrant indicates that current artificial intelligence algorithms have confirmation but limited exclusion ability (positive likelihood ratio>10 and negative likelihood ratio>0.1), supporting their role as screening adjuncts rather than definitive diagnostic tools. LRN: likelihood ratio for a negative test; LRP: likelihood ratio for a positive test.

As depicted in Figure 4, the SROC curve revealed the AUC of 0.94 (95% CI 0.92‐0.96) for AI algorithms predicting GDM, suggesting a strong predictive capability. Furthermore, we set the pretest probability at 20% based on the pretest probability of the disease. At this level, when patients were predicted to have GDM by the AI algorithms, the true positive rate was 79%, and when the prediction was not GDM, the false negative rate was 4% (Figure 5). Moreover, the model demonstrated a positive LR of 15 and a negative LR of 0.17 (Figure 5). However, the summary LR plot for the AI algorithms was located in the upper right quadrant (positive LR>10 and negative LR>0.1: confirmation only), and the individual studies were widely dispersed (Figure 6). The results indicated that while the prediction models built on AI algorithms generally demonstrated acceptable performance, they were not yet adequate for definitive diagnosis or exclusion of GDM. Additionally, there were notable variations in performance among the existing models.

### Predictors

From the models included in this systematic review, all reported predictors were systematically extracted and cataloged. The selection of key predictors for presentation and further analysis was based on three principal criteria: (1) clinical and pathophysiological relevance to GDM development, as established in prior literature and clinical guidelines; (2) frequency of reporting across the included studies, ensuring the findings were representative of common modeling practices; and (3) feasibility of meta-analytic synthesis, prioritizing variables with consistent definitions and measurements.

The consistently reported and clinically salient predictors were age, pre-pregnancy body mass index, first-trimester fast blood glucose, family history of diabetes, parity, gravidity, and history of GDM. These factors were well-recognized risk determinants in existing GDM etiological research and screening protocols. Detailed information is provided in Multimedia Appendix 4.

### Assessment of Small-Study Effects

**Figure 7.**
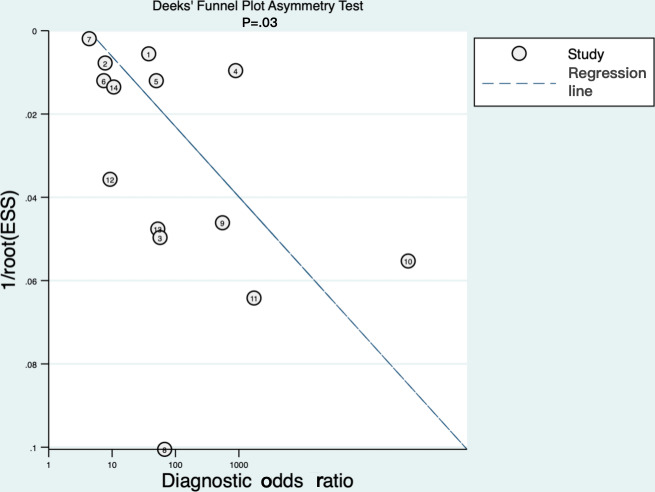
Deek funnel plot asymmetry test of small-study effects. The asymmetric distribution of studies suggests potential publication bias, where smaller studies reporting higher accuracy may be overrepresented in the literature.

Deek test (*P*=.03) and the funnel plot (Figure 7) both showed clear asymmetry, suggesting the presence of small-study effects. This asymmetry might stem from publication bias, selective reporting, and methodological differences among smaller studies.

### Heterogeneity Analysis

#### Threshold Effect Analysis

**Figure 8.**
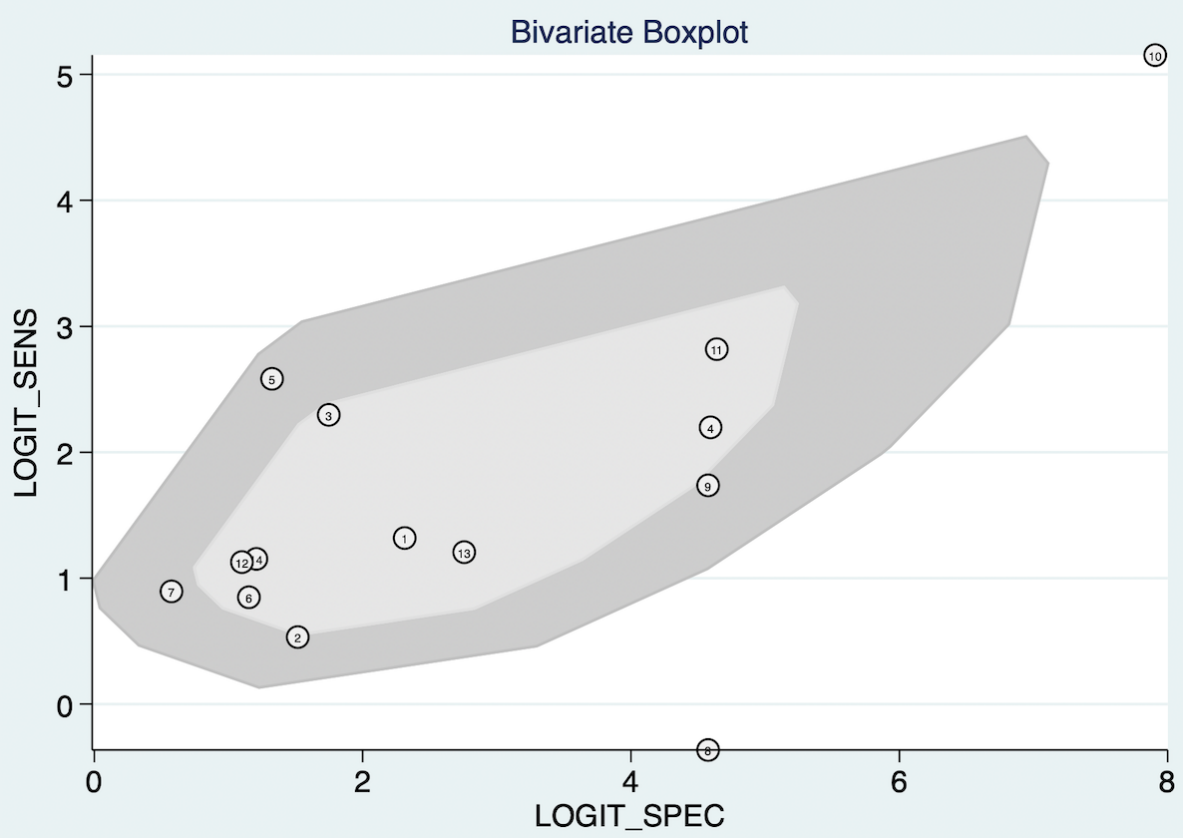
Bivariate boxplot of threshold effect analysis. This plot evaluates whether heterogeneity in results can be explained by differences in diagnostic thresholds used across studies, with dispersed points indicating substantial variability beyond what threshold effects alone can explain.

Bivariate boxplot (Figure 8) showed a positive correlation between the sensitivity and specificity of the included studies, indicating the absence of a threshold effect among the studies included in this systematic review. Moreover, some individual studies fell outside the shaded area, indicating the potential presence of heterogeneity.

#### Subgroup Analysis

**Figure 9.**
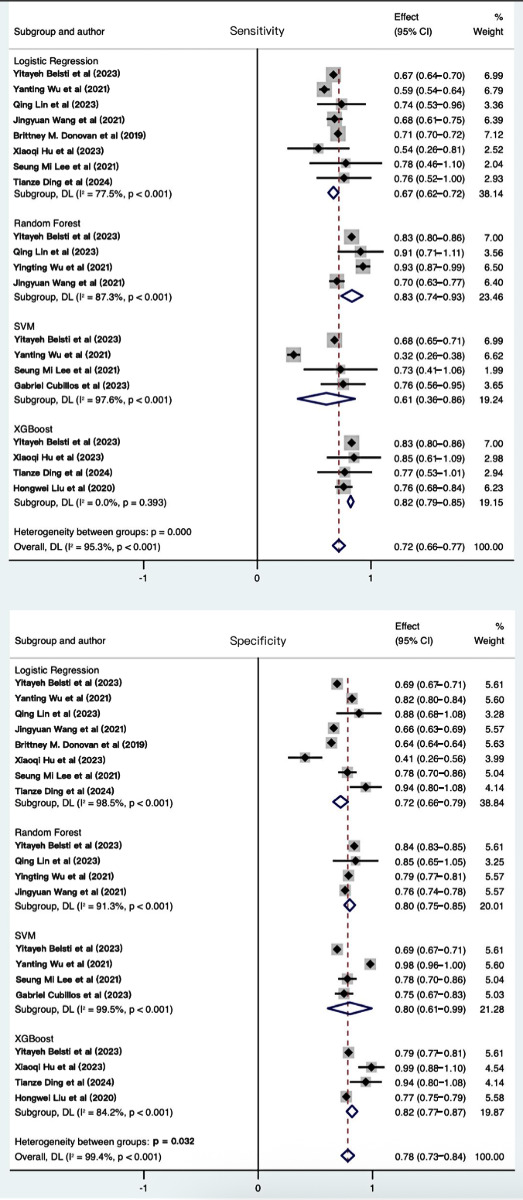
Forest plots of sensitivity and specificity in subgroup analysis. This forest plot presents pooled sensitivity and specificity estimates stratified by algorithm type (logistic regression, random forest, XGBoost, and SVM), allowing visual assessment of performance variability across model subgroups. The width of CI values reflects the precision of each estimate, while consistent point estimates across studies within a subgroup indicate algorithm-specific stability in diagnostic performance [16,21,22,28,36,38,39,41-44]. DL: deep learning; SVM: support vector machine; XGBoost: extreme gradient boosting.

To evaluate the performance of prediction models constructed using various algorithms, subgroup analyses were performed on models that had been used in at least 3 studies, after first excluding 2 studies with extreme values caused by sparse data [40,42]. The performance of each algorithm was assessed using AUC, sensitivity, specificity, positive LR, negative LR, and diagnostic odds ratio (DOR). Details are presented in Table 3 and forest plots for sensitivity and specificity are shown in Figure 9. Among the subgroup models with sparse-data studies removed, the models using the RF algorithm exhibited the highest AUC, followed by those using the XGBoost algorithm, while the models using the logistic regression algorithm demonstrated the lowest AUC performance. Additionally, these models demonstrated varying performance across different metrics. The RF algorithm exhibited the highest sensitivity (0.83, 95% CI 0.74‐0.93), while the XGBoost algorithm demonstrated the highest specificity (0.82, 95% CI 0.77‐0.87) and DOR (49, 95% CI 11‐211).

**Table 3.** Subgroup analysis of predictive performance across different artificial intelligence algorithms.

Models	Logistic regression	Random forest	XGBoost^a^	SVM^b^	*P* value
Number	8	4	4	4	—^c^
AUC^d^	0.75	0.87	0.86	0.78	<.001
Sensitivity (95% CI)	0.67 (0.62‐0.72)	0.83 (0.74‐0.93)	0.82 (0.79‐0.85)	0.61 (0.36‐0.86)	<.001
Specificity (95% CI)	0.72 (0.66‐0.79)	0.80 (0.75‐0.85)	0.82 (0.77‐0.87)	0.80 (0.61‐0.99)	.03
Positive LR^e^ (95% CI)	2.8 (1.7‐4.7)	4.5 (3.5‐5.7)	10.1 (2.9‐35.3)	4.2 (1.9‐9.2)	<.001
Negative LR (95% CI)	0.42 (0.31‐0.55)	0.17 (0.09‐0.31)	0.21 (0.16‐0.27)	0.45 (0.34‐0.60)	<.001
DOR^f^ (95% CI)	7 (3-15)	26 (12-58)	49 (11-211)	9 (5-17)	<.001

a XGBoost: extreme gradient boosting.

b SVM: support vector machine.

c Not applicable.

d AUC: area under the curve.

e LR: likelihood ratio.

f DOR: diagnostic odds ratio.

#### Meta-Regression Analysis

**Figure 10.**
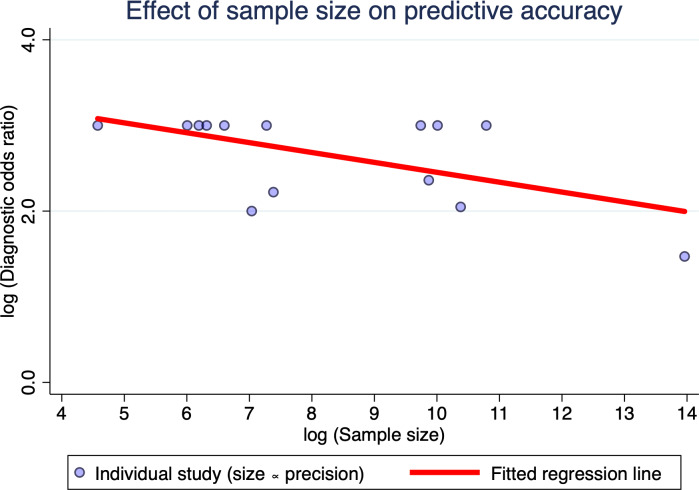
Bubble plot of meta-regression examining the association between sample size and predictive accuracy. This bubble plot visualizes the relationship between study sample size (log-transformed) and predictive accuracy (log-transformed diagnostic odds ratio) across all included studies. Each circle represents an individual study. The fitted regression line demonstrates a significant negative association, indicating that larger sample sizes tend to be associated with lower diagnostic accuracy. The plot provides an intuitive graphical confirmation of the quantitative meta-regression results, highlighting sample size as an important source of heterogeneity in model performance. log(DOR) values were truncated at ±3 for extreme cases ( fp=0 or fn=0).

To further explore the potential sources of heterogeneity in the performance of prediction models, a meta-regression analysis was conducted by including the study design (whether the study was conducted in Asia), study type (whether it was prospective), study design (whether it was multicenter), sample size (whether it exceeded 1000), GDM diagnostic criteria (whether it was based on IADPSG), and the timing of model use (whether it was in first trimester). Through meta-regression, we identified sources of heterogeneity among studies and evaluated their impact on diagnostic outcomes. The results indicated that study type significantly influenced heterogeneity among studies, with a trend toward increased predictive accuracy in prospective study designs (regression coefficient=2.289; *P*=.001). And the sample size had a substantial impact on the heterogeneity across studies, with predictive accuracy declining as the sample size increased (regression coefficient=−2.535; *P*=.001; Figure 10). This might reflect overfitting in small single-center datasets and greater clinical heterogeneity in large multicenter cohorts. Moreover, given the disparities among regions, the study area also served as one of the potential sources of heterogeneity (regression coefficient=−2.139, *P*=.002). The detailed procedures of the meta-regression are provided in Multimedia Appendix 5.

## Discussion

### Overview

This systematic review and meta-analysis aimed to evaluate the predictive performance of AI algorithms for GDM, compare the efficacy of different algorithms, and determine the key performance determinants. The pooled analysis revealed that AI-based models exhibited robust predictive capability for GDM prediction. However, the wide PIs revealed substantial performance heterogeneity in real-world applications, urging cautious interpretation of the currently summarized high-performance metrics. In addition, the summary LR plot and Fagan nomogram analyses indicated that existing models were insufficient to independently confirm or exclude GDM, so their present role should be positioned as an adjunct screening tool.

Consistent with the mainstream research trend, this systematic review further confirmed the dominant role of the RF algorithm in predicting GDM, which corroborated the findings of prior systematic reviews that highlighted ensemble methods for their robustness [14,45]. However, our analysis moved beyond merely confirming superiority by quantifying its extent and contrasting it with other algorithms. Specifically, the RF algorithm performed the best in key metrics such as AUC and sensitivity, mainly because it handles the complex, non-linear relationships inherent in GDM prediction more effectively than linear models [46,47]. This was particularly relevant in clinical settings where data could be incomplete; the inherent ability of RF to handle missing values gracefully contributed to its stronger robustness when dealing with the imperfect data often presented in routine care, which was a critical practical advantage for implementation in real-world settings [47]. In contrast, the XGBoost algorithm demonstrated higher specificity, probably benefiting from its built-in regularization and feature-importance ranking, which made it more proficient at identifying true-negative cases [48,49]. It was worth noting that the 95% CI for the DOR of XGBoost was wide, reflecting marked between-study differences in sample size, event rate, or clinical heterogeneity and indicating that its actual diagnostic consistency was highly dependent on specific population characteristics and implementation settings. Notably, this systematic review identified and emphasized methodological heterogeneity as a key driver of performance disparities. Inconsistencies across studies in data preprocessing (eg, handling of missing values and feature scaling), validation strategies (eg, data split ratios and internal validation methods), and performance reporting standards significantly hindered the comparability and integrability of research outcomes. Therefore, while pursuing superior algorithms, future studies should prioritize the establishment and adherence to methodological reporting standards for the development and validation of AI-based prediction models.

To further elucidate the real-world implications of our findings, our meta-regression analysis identified several influential factors related to variations in model accuracy, providing a more nuanced understanding than simple performance pooling. Specifically, we found that prospective study design was associated with significantly higher predictive accuracy. This might be attributed to more standardized data collection procedures and better control of confounders in prospective settings, whereas retrospective studies often relied on preexisting eHealth record data, which could be heterogeneous and incomplete [50-52]. These findings aligned with the results reported by Liu et al [53], who reported that AI-based models in prospective cohort studies achieved AUC values 4%‐7% higher than those from retrospective studies. This consistency across different analyses strengthened the argument for prioritizing prospective validation designs. Additionally, we observed that studies with larger sample sizes tended to report lower accuracy estimates. This counterintuitive finding was crucial, as it likely reflected greater demographic and clinical diversity in larger cohorts, thereby reducing overfitting and offering a more realistic, generalizable performance assessment than optimistic estimates from small, homogeneous samples. This underscored that larger, more diverse studies provided a more trustworthy evidence base for clinical deployment. Similarly, studies conducted in certain geographic regions also showed systematically lower accuracy, possibly due to regional differences in diagnostic criteria, risk factor prevalence, or health care infrastructure. These findings indicated that the performance of a model depended not only on the algorithm itself but was also profoundly shaped by the environment in which it was developed and validated. This had direct implications for implementation: a model successful in one region might not translate directly to another without adaptation and local validation.

Despite the strong performance of some algorithms, AI models still faced critical barriers to clinical deployment that should be addressed to realize their potential [54]. These included the “black-box” nature leading to limited interpretability, a persistent lack of large-scale external validation in diverse populations, and the absence of standardized interfaces for integration with existing clinical workflows—especially eHealth record systems [55,56]. To overcome these barriers, future efforts should adopt a multifaceted implementation-science approach. This entails: (1) prioritizing prospective, multicenter validation studies to generate high-grade, generalizable evidence; and (2) incorporating explainable AI techniques to enhance model interpretability and foster clinician confidence. Ultimately, realizing the full potential of AI in GDM prediction requires a concerted shift from merely developing accurate algorithms to engineering clinically viable, trustworthy, and deployable solutions.

### Limitations

However, several limitations exist in this systematic review and meta-analysis. First, most included studies and citations focused on East Asian populations, which might limit the generalizability of our findings to multi-ethnic or low-resource settings. External validation in diverse cohorts from Europe, North America, and Africa should therefore be needed to assess global applicability and to examine performance after feature-set simplification. Second, owing to limited application frequency, several emerging algorithms such as artificial neural networks and DL were not included in the subgroup analysis. Future studies should pay attention to the development of these emerging algorithms, verify their performance through more empirical studies, and explore their unique value in GDM prediction. Third, the Deek funnel plot asymmetry test indicated potential publication bias, suggesting that studies reporting higher performance metrics might be overrepresented. This could inflate the pooled estimates and limit generalizability. Future studies should consider preregistering protocols and sharing analysis code and datasets to improve reproducibility and reduce selective reporting.

### Conclusions

This systematic review and meta-analysis confirmed the strong discriminative performance of AI models for GDM prediction. However, substantial heterogeneity, publication bias, and small-study effects currently limited their readiness for direct clinical deployment. Unlike previous narrative reviews, this study innovatively provided the first direct comparative and quantitative synthesis of multiple AI algorithms in this field. This approach filled a critical gap in existing literature by offering an evidence-based framework to guide algorithm selection, rather than merely summarizing performance metrics. The key implication for real-world application was the demonstrated need for local validation in target populations before implementation. To translate this potential into practice, future studies must prioritize prospective, multicenter, large-scale external validations. The ultimate goal was to develop AI tools that were not only accurate but also interpretable and seamlessly integrable into clinical workflows, thereby enabling reliable AI-driven early prediction and management of GDM.

## Supplementary material

10.2196/79729 Multimedia Appendix 1 Details of search strategy and result.

10.2196/79729 Multimedia Appendix 2 Details of model performance parameters for each study.

10.2196/79729 Multimedia Appendix 3 Result of Prediction Model Risk of Bias Assessment Tool assessment.

10.2196/79729 Multimedia Appendix 4 Predictive factors.

10.2196/79729 Multimedia Appendix 5 Result of meta-regression.

10.2196/79729 Checklist 1 PRISMA checklists.

## Abbreviations

AI: artificial intelligence
AUC: area under the curve
DL: deep learning
DOR: diagnostic odds ratio
GDM: gestational diabetes mellitus
LR: likelihood ratio
ML: machine learning
PI: prediction interval
PRISMA: Preferred Reporting Items for Systematic Reviews and Meta-Analysis
PROBAST: Prediction Model Risk of Bias Assessment Tool
RF: random forest
ROB: risk of bias
SROC: summary receiver operating characteristic curve
XGBoost: extreme gradient boosting
τ: standard deviation
τ²: standard variance
