# Supplementary material for: Predictive Performance of Artificial Intelligence Algorithms for Gestational Diabetes Mellitus in Pregnant Women: Systematic Review and Meta-Analysis
Source: J Med Internet Res. 2026 Jan 30;28:e79729. doi: 10.2196/79729 (PMC12858046; doi:10.2196/79729)
Supplement: Multimedia Appendix 1 [file jmir-v28-e79729-s001.docx]

Search strategy used for the electronic databases

**PubMed database search: 225 results**

| No. | Query | Results |
| --- | --- | --- |
| 1 | ((((((((((Diabetes, Gestational[MeSH Terms]) OR (Diabetes Mellitus, Gestational[Title/Abstract])) OR (Gestational Diabetes Mellitus[Title/Abstract])) OR (Diabetes, Pregnancy-Induced[Title/Abstract])) OR (Diabetes, Pregnancy Induced[Title/Abstract])) OR (Pregnancy-Induced Diabetes[Title/Abstract])) OR (Pregnancy Induced Diabetes[Title/Abstract])) OR (Gestational Diabetes[Title/Abstract])) OR (GDM[Title/Abstract])) OR (Pregnancy Diabetes Mellitus[Title/Abstract])) OR (Diabetes mellitus, gestational[Title/Abstract]) | 29,935 |
| 2 | **((((((((((((((((((((((((((((((((((Artificial Intelligence[MeSH Terms]) OR (Deep Learning[MeSH Terms])) OR (Machine Learning[MeSH Terms])) OR (Intelligence, Artificial[Title/Abstract])) OR (Computer Reasoning[Title/Abstract])) OR (Reasoning, Computer[Title/Abstract])) OR (AI (Artificial Intelligence[Title/Abstract]))) OR (Machine Intelligence[Title/Abstract])) OR (Intelligence, Machine[Title/Abstract])) OR (Computational Intelligence[Title/Abstract])) OR (Intelligence, Computational[Title/Abstract])) OR (Computer Vision Systems[Title/Abstract])) OR (Computer Vision System[Title/Abstract])) OR (System, Computer Vision[Title/Abstract])) OR (Systems, Computer Vision[Title/Abstract])) OR (Vision System, Computer[Title/Abstract])) OR (Vision Systems, Computer[Title/Abstract])) OR (Knowledge Acquisition (Computer[Title/Abstract]))) OR (Acquisition, Knowledge (Computer[Title/Abstract]))) OR (Knowledge Representation (Computer[Title/Abstract]))) OR (Knowledge Representations (Computer[Title/Abstract]))) OR (Representation, Knowledge (Computer[Title/Abstract]))) OR (Learning, Deep[Title/Abstract])) OR (Hierarchical Learning[Title/Abstract])) OR (Learning, Hierarchical[Title/Abstract])) OR (Learning, Machine[Title/Abstract])) OR (Transfer Learning[Title/Abstract])) OR (Learning, Transfer[Title/Abstract])) OR (Ensemble Learnings[Title/Abstract])) OR (Boosting Machine Learning Algorithms[Title/Abstract])) OR (Supervised Machine Learning[Title/Abstract])) OR (Support Vector Machine[Title/Abstract])) OR (Computer Neural Networks[Title/Abstract])) OR (Decision Tree[Title/Abstract])) OR (Random Forest[Title/Abstract])** | 310,278 |
| 3 | #1 and #2 | 226 |

**2.2 Web of Science(WOS) database search: 691 results**

| No. | Query | Results |
| --- | --- | --- |
| 1 | **((((((((((TS=(Diabetes, Gestational)) OR TS=(Diabetes Mellitus, Gestational)) OR TS=(Gestational Diabetes Mellitus)) OR TS=(Diabetes, Pregnancy-Induced)) OR TS=(Diabetes, Pregnancy Induced)) OR TS=(Pregnancy-Induced Diabetes)) OR TS=(Pregnancy Induced Diabetes)) OR TS=(Gestational Diabetes)) OR TS=(GDM)) OR TS=(Pregnancy Diabetes Mellitus)) OR TS=(Diabetes mellitus, gestational)** | 65,524 |
| 2 | **((((((((((((((((((((((((((((((((((TS=(Artificial Intelligence)) OR TS=(Deep Learning)) OR TS=(Machine Learning)) OR TS=(Intelligence, Artificial)) OR TS=(Computer Reasoning)) OR TS=(Reasoning, Computer)) OR TS=(AI (Artificial Intelligence))) OR TS=(Machine Intelligence)) OR TS=(Intelligence, Machine)) OR TS=(Computational Intelligence)) OR TS=(Intelligence, Computational)) OR TS=(Computer Vision Systems)) OR TS=(Computer Vision System)) OR TS=(System, Computer Vision)) OR TS=(Systems, Computer Vision)) OR TS=(Vision System, Computer)) OR TS=(Vision Systems, Computer)) OR TS=(Knowledge Acquisition (Computer))) OR TS=(Acquisition, Knowledge (Computer))) OR TS=(Knowledge Representation (Computer))) OR TS=(Knowledge Representations (Computer))) OR TS=(Representation, Knowledge (Computer))) OR TS=(Learning, Deep)) OR TS=(Hierarchical Learning)) OR TS=(Learning, Hierarchical)) OR TS=(Learning, Machine)) OR TS=(Transfer Learning)) OR TS=(Learning, Transfer)) OR TS=(Ensemble Learnings)) OR TS=(Boosting Machine Learning Algorithms)) OR TS=(Supervised Machine Learning)) OR TS=(Support Vector Machine)) OR TS=(Computer Neural Networks)) OR TS=(Decision Tree)) OR TS=(Random Forest)** | 1,547,946 |
| 3 | #1 and #2 | 692 |

**2.3 Cochrane Library database search: 338 results**

| No. | Query | Results |
| --- | --- | --- |
| 1 | **MeSH descriptor: [Diabetes, Gestational] explode all trees** | 1,693 |
| 2 | **Diabetes Mellitus, Gestational** | 3,441 |
| 3 | **Gestational Diabetes Mellitus** | 3,441 |
| 4 | Diabetes, Pregnancy-Induced | 313 |
| 5 | Diabetes, Pregnancy Induced | 708 |
| 6 | Pregnancy-Induced Diabetes | 313 |
| 7 | Pregnancy Induced Diabetes | 708 |
| 8 | Gestational Diabetes | 5,078 |
| 9 | GDM | 2,069 |
| 10 | Pregnancy Diabetes Mellitus | 4,532 |
| 11 | Diabetes mellitus, gestational | 3,441 |
| 12 | #1 or #2 or #3 or #4 or #5 or #6 or #7 or #8 or #9 or #10 or #11 | 7,002 |
| 13 | MeSH descriptor: [Artificial Intelligence] explode all trees | 3,388 |
| 14 | MeSH descriptor: [Deep Learning] explode all trees | 356 |
| 15 | MeSH descriptor: [Machine Learning] explode all trees | 1,082 |
| 16 | Intelligence, Artificial | 2,797 |
| 17 | Computer Reasoning | 392 |
| 18 | Reasoning, Computer | 392 |
| 19 | AI (Artificial Intelligence) | 1,267 |
| 20 | Machine Intelligence | 509 |
| 21 | Intelligence, Machine | 509 |
| 22 | Computational Intelligence | 131 |
| 23 | Intelligence, Computational | 131 |
| 24 | Computer Vision Systems | 336 |
| 25 | Computer Vision System | 787 |
| 26 | System, Computer Vision | 787 |
| 27 | Systems, Computer Vision | 336 |
| 28 | Vision System, Computer | 787 |
| 29 | Vision Systems, Computer | 336 |
| 30 | Knowledge Acquisition (Computer) | 469 |
| 31 | Acquisition, Knowledge (Computer) | 469 |
| 32 | Knowledge Representation (Computer) | 280 |
| 33 | Knowledge Representations (Computer) | 62 |
| 34 | Representation, Knowledge (Computer) | 280 |
| 35 | Learning, Deep | 1,898 |
| 36 | Hierarchical Learning | 403 |
| 37 | Learning, Hierarchical | 403 |
| 38 | Learning, Machine | 3,573 |
| 39 | Transfer Learning | 1,992 |
| 40 | Learning, Transfer | 1,992 |
| 41 | Ensemble Learnings | 0 |
| 42 | Boosting Machine Learning Algorithms | 77 |
| 43 | Supervised Machine Learning | 227 |
| 44 | Support Vector Machine | 585 |
| 45 | Computer Neural Networks | 604 |
| 46 | Decision Tree | 1,241 |
| 47 | Random Forest | 7,819 |
| 48 | #13 or #14 or #15 or #16 or #17 or #18 or #19 or #20 or #21 or #22 or #23 or #24 or #25 or #26 or #27 or #28 or #29 or #30 or #31 or #32 or #33 or #34 or #35 or #36 or #37 or #38 or #39 or #40 or #41 or #42 or #43 or #44 or #45 or #46 or #47 | 19,926 |
| 49 | #12 and #48 | 338 |

**2.4 Embase database search: 553 results**

| No. | Query | Results |
| --- | --- | --- |
| 1 | 'diabetes, gestational'/exp OR 'diabetes,gestational' OR 'gestational diabetes mellitus'/exp OR 'gestational diabetes mellitus' OR 'diabetes pregnancy induced' OR 'pregnancy induced diabetes'/exp OR 'pregnancy induced diabetes' OR 'gestational diabetes'/exp OR 'gestational diabetes' OR 'gdm' OR 'pregnancy diabetes mellitus'/exp OR 'pregnancy diabetes mellitus' OR 'diabetes mellitus gestational' | 63,028 |
| 2 | 'artificial intelligence' OR 'deep learning' OR 'machine learning' OR 'intelligence, artificial' OR 'computer reasoning' OR 'reasoning, computer' OR 'ai (artificial intelligence)' OR 'machine intelligence' OR 'intelligence, machine' OR 'computational intelligence' OR 'intelligence, computational' OR 'computer vision systems' OR 'computer vision system' OR 'system, computer vision' OR 'systems, computer vision' OR 'vision system, computer' OR 'vision systems, computer' OR 'knowledge acquisition (computer)' OR 'acquisition, knowledge (computer)' OR 'knowledge representation (computer)' OR 'knowledge representations (computer)' OR 'representation, knowledge (computer)' OR 'learning, deep' OR 'hierarchical learning' OR 'learning, hierarchical' OR 'learning, machine' OR 'transfer learning' OR 'learning, transfer' OR 'ensemble learnings' OR 'boosting machine learning algorithms' OR 'supervised machine learning' OR 'support vector machine' OR 'computer neural networks' OR 'decision tree' OR 'random forest' | 426,658 |
| 3 | #1 and #2 | 553 |

**2.5 Scopus database search: 744 results**

| No. | Query | Results |
| --- | --- | --- |
| 1 | ( TITLE-ABS-KEY ( "Diabetes, Gestational" or "Diabetes Mellitus, Gestational" or "Gestational Diabetes Mellitus" or "Diabetes, Pregnancy-Induced" or "Diabetes, Pregnancy Induced" or "Pregnancy-Induced Diabetes" or "Pregnancy Induced Diabetes" or "Gestational Diabetes" or "GDM" or "Pregnancy Diabetes Mellitus" or "Diabetes mellitus, gestational" ) ) AND ( TITLE-ABS-KEY  ( "artificial intelligence" OR "deep learning" OR "machine learning" OR "intelligence, artificial" OR "computer reasoning" OR "reasoning, computer" OR "ai (artificial intelligence)" OR "machine intelligence" OR "intelligence, machine" OR "computational intelligence" OR"intelligence, computational" OR "computer vision systems" OR "computer vision system" OR "system, computer vision" OR "systems, computer vision" OR "vision system, computer" OR "vision systems, computer" OR"knowledge acquisition (computer)" OR "acquisition, knowledge (computer)" OR "knowledge representation (computer)" OR "knowledge representations (computer)" OR "representation, knowledge (computer)" OR"learning, deep" OR "hierarchical learning" OR "learning, hierarchical" OR "learning, machine" OR "transfer learning" OR "learning, transfer" OR "ensemble learnings" OR "boosting machine learning algorithms" OR"supervised machine learning" OR "support vector machine" OR "computer neural networks" OR "decision tree"OR "random forest" ) ) | 746 |

**2.6 OVID database search: 239 results**

| No. | Query | Results |
| --- | --- | --- |
| 1 | ("Diabetes, Gestational" or "Diabetes Mellitus, Gestational" or "Gestational Diabetes Mellitus" or "Diabetes, Pregnancy-Induced" or "Diabetes, Pregnancy Induced" or "Pregnancy-Induced Diabetes" or "Pregnancy Induced Diabetes" or "Gestational Diabetes" or "GDM" or "Pregnancy Diabetes Mellitus" or "Diabetes mellitus, gestational").mp. [mp=title, book title, abstract, original title, name of substance word, subject heading word, floating sub-heading word, keyword heading word, organism supplementary concept word, protocol supplementary concept word, rare disease supplementary concept word, unique identifier, synonyms, population supplementary concept word, anatomy supplementary concept word] | 26,315 |
| 2 | **(**“artificial intelligence” OR “deep learning” OR “machine learning” OR “intelligence, artificial” OR “computer reasoning” OR “reasoning, computer” OR “AI (artificial intelligence)” OR “machine intelligence” OR “intelligence, machine” OR “computational intelligence” OR “intelligence, computational” OR “computer vision systems” OR “computer vision system” OR “system, computer vision” OR “systems, computer vision” OR “vision system, computer” OR “vision systems, computer” OR “knowledge acquisition (computer)” OR “acquisition, knowledge (computer)” OR “knowledge representation (computer)” OR “knowledge representations (computer)” OR “representation, knowledge (computer)” OR “learning, deep” OR “hierarchical learning” OR “learning, hierarchical” OR “learning, machine” OR “transfer learning” OR “learning, transfer” OR “ensemble learnings” OR “boosting machine learning algorithms” OR “supervised machine learning” OR “support vector machine” OR “computer neural networks” OR “decision tree” OR “random forest”**).mp. [mp=title, book title, abstract, original title, name of substance word, subject heading word, floating sub-heading word, keyword heading word, organism supplementary concept word, protocol supplementary concept word, rare disease supplementary concept word, unique identifier, synonyms, population supplementary concept word, anatomy supplementary concept word]** | 304,838 |
| 3 | #1 and #2 | 240 |
