# Supplementary material for: Predictive Performance of Artificial Intelligence Algorithms for Gestational Diabetes Mellitus in Pregnant Women: Systematic Review and Meta-Analysis
Source: J Med Internet Res. 2026 Jan 30;28:e79729. doi: 10.2196/79729 (PMC12858046; doi:10.2196/79729)
Supplement: Multimedia Appendix 2 [file jmir-v28-e79729-s002.docx]

**Table S1.**

| **Author, Year** | **ML/DL algorithm** | **Optimal model** | **AUC** | **Accuracy** | **Sensitivity** | **Specificity** |
| --- | --- | --- | --- | --- | --- | --- |
| Belsti et al  (2023) [22] | LR,KNN,GNB,SVM,MLP,  DT,RF,ExtRaTrees,AdaBoost,  GBM,CatBoost,XGBoost | CatBoost | LR=75%,KNN=86%,GNB=71%,SVM=75%,MLP=82%,DT=79%,RF=92%,ExtRaTrees=79%,  AdaBoost=90%,GBM=91%,  CatBoost=92%,XGBoost=92% | LR=69%,KNN=80%,GNB=59%,  SVM=69%,MLP=75%,DT=78%,  RF=84%,ExtRaTrees=72%,  AdaBoost=81%,GBM=84%,  CatBoost=84%,XGBoost=84% | LR=67%,KNN=87%,  GNB=56%,SVM=68%,  MLP=77%,DT=76%,  RF=83%,ExtRaTrees=71%,  AdaBoost=77%,GBM=83%,  CatBoost=79%,  XGBoost=83% | LR=69%,KNN=75%,  GNB=76%,SVM=69%,  MLP=73%,DT=79%,  RF=84%,ExtRaTrees=73%,  AdaBoost=86%,GBM=85%,  CatBoost=91%,  XGBoost=79% |
| Ali et al  (2022) [41] | RF,GBM,XGBoost | XGBoost | XGBoost=77% | - | - | - |
| Wu et al  (2021) [16] | LR,KNN,SVM,DNN | DNN | LR=77%,KNN=65%,  SVM=77%,DNN=80% | - | LR=59%,KNN=31%,  SVM=32%,DNN=63% | LR=82%,KNN=98%,  SVM=98%,DNN=82% |
| Qing Lin and Fang  (2023) [28] | LR,RF | RF | LR=88%,RF=95% | - | LR=74%,RF=91% | LR=88%,RF=85% |
| Ye et al  (2020) [29] | LR,GBDT,AdaBoost,LGB,  Logistic,Vote,XGB,DT,RF | GBDT | LR=71%,GBDT=75% | - | GBDT=90% | GBDT=99% |
| Wang et al  (2022) [38] | LR,DT,RF | DT | LR=89%,DT=83%,RF=82% | DT=77%,RF=76% | - | DT=83%,RF=79% |
| Wu et al  (2021) [30] | LR,RF | RF | LR=70%,RF=75% | - | RF=93% | RF=79% |
| Wang et al  (2021) [31] | LR,RF | RF | LR=69%,RF=75% | LR=66%,RF=76% | LR=68%,RF=70% | LR=66%,RF=76% |
| Syngelaki et al  (2025) [39] | LR | LR | LR=76% | - | - | - |
| Donovan et al  (2019) [32] | LR | LR | LR=73% | - | LR=71% | LR=64% |
| Kaya et al  (2024) [33] | XGBoost | XGBoost | XGBoost=73% | XGBoost=73% | XGBoost=40% | XGBoost=100% |
| Hu et al  (2023) [34] | LR,XGBoost | XGBoost | LR=75%,XGBoost=94% | LR=79%,XGBoost=88% | LR=54%,XGBoost=85% | LR=41%,XGBoost=99% |
| Liu et al  (2022) [42] | LR,RF,XGBoost | XGBoost | XGBoost=62% | RF=64%,  LR+RF+XGBoost=67% | XGBoost=60% | RF=68%,  LR+RF+XGBoost=73% |
| Lee et al  (2021) [35] | LR,RF,SVM,DNN | RF | LR=84%,RF=100%,  SVM=80%,DNN=81% | - | LR=78%,RF=100%,  SVM=73%,DNN=76% | LR=78%,RF=100%,  SVM=78%,DNN=68% |
| Kumar et al  (2022) [43] | GBM+SVM | GBM+SVM | GBM+SVM=93% | - | - | - |
| Bigdeli et al  (2025) [] | DT,MLP,KNN,GNB,RF,  XGBoost | RF | DT=82%,MLP=74%,  KNN=77%,GNB=72%,  RF=94%,XGBoost=88% | RF=89% | - | - |
| Kurt et al  (2023) [15] | RNN-LSTM | RNN-LSTM | RNN-LSTM=98% | RNN-LSTM=98% | RNN-LSTM=95% | RNN-LSTM=99% |
| Cubillos et al  (2023) [21] | MLP,SVM | SVM | MLP=81%,SVM=81% | MLP=71%,SVM=76% | MLP=85%,SVM=82% | MLP=69%,SVM=75% |
| Ding et al  (2024) [36] | LR,XGBoost,LightBoost | XGBoost | LR=71%,XGBoost=79%,  LightBoost=75% | LR=76%,XGBoost=77%,  LightBoost=76% | LR=27%,XGBoost=32%,  LightBoost=36% | LR=94%,XGBoost=94%,  LightBoost=92% |
| Kang et al  (2023) [37] | XGBoost | XGBoost | XGBoost=88% | - | - | - |
| Zhao et al  (2025) [40] | MLP,MLP_NM | MLP_NM | MLP=77%,MLP_NM=95% | MLP=87%,  MLP_NM=89% | - | - |
| Liu et al  (2020) [44] | XGBoost | XGBoost | XGBoost=74% | XGBoost=76% | XGBoost=62% | XGBoost=77% |

### **References**

15. Kurt B, Gürlek B, Keskin S, et al. Prediction of gestational diabetes using deep learning and Bayesian optimization and traditional machine learning techniques. Med Biol Eng Comput. Jul 2023;61(7):1649-1660. [doi: ] [Medline: 36848010]

16. Wu YT, Zhang CJ, Mol BW, et al. Early prediction of gestational diabetes mellitus in the Chinese population via advanced machine learning. J Clin Endocrinol Metab. Mar 8, 2021;106(3):e1191-e1205. [doi: ] [Medline: 33351102]

21. Cubillos G, Monckeberg M, Plaza A, et al. Development of machine learning models to predict gestational diabetes risk in the first half of pregnancy. BMC Pregnancy Childbirth. Jun 23, 2023;23(1):469. [doi: ] [Medline: 37353749]

22. Belsti Y, Moran L, Du L, et al. Comparison of machine learning and conventional logistic regression-based prediction models for gestational diabetes in an ethnically diverse population; the Monash GDM Machine learning model. Int J Med Inform. Nov 2023;179:105228. [doi: ] [Medline: 37774429]

28. Lin Q, Fang ZJ. Establishment and evaluation of a risk prediction model for gestational diabetes mellitus. World J Diabetes. Oct 15, 2023;14(10):1541-1550. [doi: ] [Medline: 37970129]

29. Ye Y, Xiong Y, Zhou Q, Wu J, Li X, Xiao X. Comparison of machine learning methods and conventional logistic regressions for predicting gestational diabetes using routine clinical data: a retrospective cohort study. J Diabetes Res. 2020;2020:4168340. [doi: ] [Medline: 32626780]

30. Wu Y, Ma S, Wang Y, et al. A risk prediction model of gestational diabetes mellitus before 16 gestational weeks in Chinese pregnant women. Diabetes Res Clin Pract. Sep 2021;179:109001. [doi: ] [Medline: 34390760]

31. Wang J, Lv B, Chen X, et al. An early model to predict the risk of gestational diabetes mellitus in the absence of blood examination indexes: application in primary health care centres. BMC Pregnancy Childbirth. Dec 8, 2021;21(1):814. [doi: ] [Medline: 34879850]

32. Donovan BM, Breheny PJ, Robinson JG, et al. Development and validation of a clinical model for preconception and early pregnancy risk prediction of gestational diabetes mellitus in nulliparous women. PLoS ONE. 2019;14(4):e0215173. [doi: ] [Medline: 30978258]

33. Kaya Y, Bütün Z, Çelik Ö, Salik EA, Tahta T, Yavuz AA. The early prediction of gestational diabetes mellitus by machine learning models. BMC Pregnancy Childbirth. Aug 31, 2024;24(1):574. [doi: ] [Medline: 39217284]

34. Hu X, Hu X, Yu Y, Wang J. Prediction model for gestational diabetes mellitus using the XG Boost machine learning algorithm. Front Endocrinol (Lausanne). 2023;14:1105062. [doi: ] [Medline: 36967760]

35. Lee SM, Hwangbo S, Norwitz ER, et al. Nonalcoholic fatty liver disease and early prediction of gestational diabetes mellitus using machine learning methods. Clin Mol Hepatol. Jan 2022;28(1):105-116. [doi: ] [Medline: 34649307]

36. Ding T, Liu P, Jia J, Wu H, Zhu J, Yang K. Application of machine learning algorithm incorporating dietary intake in prediction of gestational diabetes mellitus. Endocr Connect. Dec 1, 2024;13(12):e240169. [doi: ] [Medline: 39393404]

37. Kang BS, Lee SU, Hong S, et al. Prediction of gestational diabetes mellitus in Asian women using machine learning algorithms. Sci Rep. Aug 16, 2023;13(1):13356. [doi: ] [Medline: 37587201]

38. Wang N, Guo H, Jing Y, et al. Development and validation of risk prediction models for gestational diabetes mellitus using four different methods. Metabolites. Oct 29, 2022;12(11):1040. [doi: ] [Medline: 36355123]

39. Syngelaki A, Wright A, Gomez Fernandez C, Mitsigiorgi R, Nicolaides KH. First-trimester prediction of gestational diabetes mellitus based on maternal risk factors. BJOG. Jun 2025;132(7):972-982. [doi: ] [Medline: 40000426]

40. Zhao M, Su X, Huang L. Early gestational diabetes mellitus risk predictor using neural network with NearMiss. Gynecol Endocrinol. Dec 2025;41(1):2470317. [doi: ] [Medline: 39992231]

41. Ali N, Khan W, Ahmad A, Masud MM, Adam H, Ahmed LA. Predictive modeling for the diagnosis of gestational diabetes mellitus using epidemiological data in the United Arab Emirates. Information. 2022;13(10):485. [doi: ]

42. Liu R, Zhan Y, Liu X, et al. Stacking ensemble method for gestational diabetes mellitus prediction in Chinese pregnant women: a prospective cohort study. J Healthc Eng. 2022;2022:8948082. [doi: ] [Medline: 36147870]

43. Kumar M, Ang LT, Png H, et al. Automated machine learning (AutoML)-derived preconception predictive risk model to guide early intervention for gestational diabetes mellitus. Int J Environ Res Public Health. Jun 1, 2022;19(11):6792. [doi: ] [Medline: 35682375]

44. Liu H, Li J, Leng J, et al. Machine learning risk score for prediction of gestational diabetes in early pregnancy in Tianjin, China. Diabetes Metab Res Rev. Jul 2021;37(5):e3397. [doi: ] [Medline: 32845061]

Kianian, et al
