# Supplementary material for: Predictive Performance of Artificial Intelligence Algorithms for Gestational Diabetes Mellitus in Pregnant Women: Systematic Review and Meta-Analysis
Source: J Med Internet Res. 2026 Jan 30;28:e79729. doi: 10.2196/79729 (PMC12858046; doi:10.2196/79729)
Supplement: Multimedia Appendix 3 [file jmir-v28-e79729-s003.docx]

**Figure S1.** Summary of the risk of bias for each study. Green, yellow, and red circles indicate low, unclear, and high risk of bias, respectively.


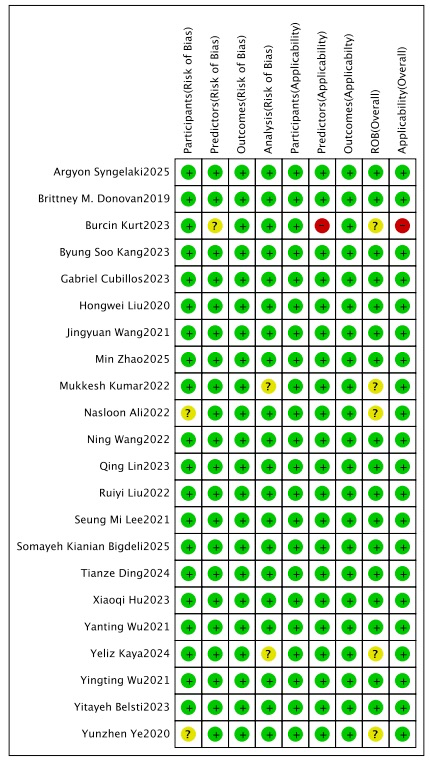


**Table S1.** Item-level justifications for ‘Unclear’ risk of bias judgments in PROBAST assessment

| Author, Year | PROBAST Domain | Specific Item | Judgment | Justification |
| --- | --- | --- | --- | --- |
| Ali et al  (2022) [41] | Participants | 1.3 Did the inclusion and exclusion of participants result in a representative dataset? | Unclear | The study described the data source and study design. However, no in-depth rationale for the inclusion and exclusion criteria was provided. |
| Ye et al  (2020) [29] | Participants | 1.3 Did the inclusion and exclusion of participants result in a representative dataset? | Unclear | The study described the data source and study design, but did not provide a detailed explanation of the inclusion and exclusion process. |
| Kurt et al  (2023) [15] | Predictors | 2.3 Was the processing of predictors not based on the outcome data? | Unclear | The study only reported the number and source of the predictors but did not provide a detailed explanation of their processing. |
| Kumar et al (2022) [43] | Analysis | 4.1 Was there evidence that the sample size was adequate? | Unclear | No details on the sample size calculation were provided. |
| Kaya et al  (2024) [33] | Analysis | 4.1 Was there evidence that the sample size was adequate? | Unclear | No details on the sample size calculation were provided. |

### **References**

15. Kurt B, Gürlek B, Keskin S, et al. Prediction of gestational diabetes using deep learning and Bayesian optimization and traditional machine learning techniques. Med Biol Eng Comput. Jul 2023;61(7):1649-1660. [doi: ] [Medline: 36848010]

29. Ye Y, Xiong Y, Zhou Q, Wu J, Li X, Xiao X. Comparison of machine learning methods and conventional logistic regressions for predicting gestational diabetes using routine clinical data: a retrospective cohort study. J Diabetes Res. 2020;2020:4168340. [doi: ] [Medline: 32626780]

33. Kaya Y, Bütün Z, Çelik Ö, Salik EA, Tahta T, Yavuz AA. The early prediction of gestational diabetes mellitus by machine learning models. BMC Pregnancy Childbirth. Aug 31, 2024;24(1):574. [doi: ] [Medline: 39217284]

41. Ali N, Khan W, Ahmad A, Masud MM, Adam H, Ahmed LA. Predictive modeling for the diagnosis of gestational diabetes mellitus using epidemiological data in the United Arab Emirates. Information. 2022;13(10):485. [doi: ]

43. Kumar M, Ang LT, Png H, et al. Automated machine learning (AutoML)-derived preconception predictive risk model to guide early intervention for gestational diabetes mellitus. Int J Environ Res Public Health. Jun 1, 2022;19(11):6792. [doi: ] [Medline: 35682375]
