# Supplementary material for: Predictive Performance of Artificial Intelligence Algorithms for Gestational Diabetes Mellitus in Pregnant Women: Systematic Review and Meta-Analysis
Source: J Med Internet Res. 2026 Jan 30;28:e79729. doi: 10.2196/79729 (PMC12858046; doi:10.2196/79729)
Supplement: Multimedia Appendix 4 [file jmir-v28-e79729-s004.docx]

**Table S2.**

| No | factors | Number | No | factors | Number |
| --- | --- | --- | --- | --- | --- |
| 1 | Age | 17 | 24 | First-trimester abdomen circumference | 2 |
| 2 | Pre-pregnancy body mass index | 16 | 25 | ALT | 2 |
| 3 | First-trimester fast blood glucose | 16 | 26 | Height | 1 |
| 4 | Weight | 11 | 27 | Education attainment | 1 |
| 5 | Family history of diabetes | 7 | 28 | Mean arterial pressure | 1 |
| 6 | Parity | 7 | 29 | History of macrosomia | 1 |
| 7 | Gravidity | 7 | 30 | History of poor obstetric outcomes | 1 |
| 8 | Race | 6 | 31 | Hemoglobin | 1 |
| 9 | Hypertension | 5 | 32 | Serum ferritin | 1 |
| 10 | History of gestational diabetes mellitus | 5 | 33 | Irregular menstruation | 1 |
| 11 | First-trimester triglyceride | 5 | 34 | Previous birth weight | 1 |
| 12 | Use of anti-hypertension drugs | 5 | 35 | Smoking status | 1 |
| 13 | Inflammatory bowel disease | 4 | 36 | Hepatic steatosis index | 1 |
| 14 | Cardiovascular disease | 3 | 37 | NAFLD | 1 |
| 15 | Chronic kidney disease | 3 | 38 | Physical inactivity | 1 |
| 16 | First-trimester HbA1c | 3 | 39 | Intake of livestock meat | 1 |
| 17 | Use of illicit drugs | 3 | 40 | Intake of aquatic products | 1 |
| 18 | First-trimester HDL | 2 | 41 | Selenium | 1 |
| 19 | PCOS | 2 | 42 | SBP | 1 |
| 20 | Psychiatric disorders | 2 | 43 | DBP | 1 |
| 21 | Insulin resistance | 2 | 44 | Intake of grain | 1 |
| 22 | Urinal tract diseases | 2 | 45 | Income | 1 |
| 23 | Intake of VitE | 2 | 46 | Hip circumference | 1 |
