# Supplementary material for: Predictive Performance of Artificial Intelligence Algorithms for Gestational Diabetes Mellitus in Pregnant Women: Systematic Review and Meta-Analysis
Source: J Med Internet Res. 2026 Jan 30;28:e79729. doi: 10.2196/79729 (PMC12858046; doi:10.2196/79729)
Supplement: Multimedia Appendix 5 [file jmir-v28-e79729-s005.docx]

In this study, meta-regression was employed to examine the relationship between study-level characteristic and effect sizes in order to explore potential sources of heterogeneity. This approach extended traditional meta-analysis by incorporating covariates that might influence the observed outcomes.

**Table S3.**

| Variety | Coeff. | Std. Err. | p – value | RDOR | [95%CI] |
| --- | --- | --- | --- | --- | --- |
| Cte. | 11.809 | 2.0202 | 0.0000 | ---- | ---- |
| S | -0.777 | 0.1567 | 0.0000 | ---- | ---- |
| Study region | -1.890 | 0.9578 | 0.0580 | 0.15 | (0.02;1.07) |
| Study type | 1.938 | 1.0501 | 0.0752 | 6.94 | (0.81;59.46) |
| Study design | 0.309 | 0.7825 | 0.6962 | 1.36 | (0.27;6.75) |
| Diagnostic criteria | -0.202 | 0.6406 | 0.7547 | 0.82 | (0.22;3.03) |
| Sample size | -2.563 | 0.6037 | 0.0002 | 0.08 | (0.02;0.27) |
|  |  |  |  |  |  |
| Variety | Coeff. | Std. Err. | p – value | RDOR | [95%CI] |
| Cte. | 9.303 | 1.4606 | 0.0000 | ---- | ---- |
| S | -0.769 | 0.1521 | 0.0000 | ---- | ---- |
| Study region | -1.856 | 0.9303 | 0.0552 | 0.16 | (0.02;1.05) |
| Study type | 1.971 | 1.0110 | 0.0607 | 7.18 | (0.91;56.57) |
| Study design | 0.326 | 0.7605 | 0.6715 | 1.38 | (0.29;6.55) |
| Sample size | -2.535 | 0.5845 | 0.0002 | 0.08 | (0.02;0.26) |

| Variety | Coeff. | Std. Err. | p – value | RDOR | [95%CI] |
| --- | --- | --- | --- | --- | --- |
| Cte. | 9.587 | 1.2738 | 0.0000 | ---- | ---- |
| S | -0.769 | 0.1498 | 0.0000 | ---- | ---- |
| Study region | -2.139 | 0.6307 | 0.0019 | 0.12 | (0.03;0.43) |
| Study type | 2.289 | 0.6432 | 0.0012 | 9.86 | (2.66;36.62) |
| Sample size | -2.535 | 0.5759 | 0.0001 | 0.08 | (0.02;0.26) |
